# Soft Tissue Hybrid Model for Real-Time Simulations

**DOI:** 10.3390/polym14071407

**Published:** 2022-03-30

**Authors:** Mario R. Moreno-Guerra, Oscar Martínez-Romero, Luis Manuel Palacios-Pineda, Daniel Olvera-Trejo, José A. Diaz-Elizondo, Eduardo Flores-Villalba, Jorge V. L. da Silva, Alex Elías-Zúñiga, Ciro A. Rodriguez

**Affiliations:** 1 Mechanical Engineering and Advanced Materials Department, School of Engineering and Science, Tecnologico de Monterrey, Ave. Eugenio Garza Sada 2501 Sur, Monterrey 64849, NL, Mexico; marioregino@hotmail.com (M.R.M.-G.); oscar.martinez@tec.mx (O.M.-R.); daniel.olvera.trejo@tec.mx (D.O.-T.); 2 Laboratorio Nacional de Manufactura Aditiva y Digital MADIT, Apodaca 66629, NL, Mexico; 3 Tecnológico Nacional de Mexico, Instituto Tecnológico de Pachuca, Carr. México-Pachuca Km 87.5, Pachuca de Soto 42080, HG, Mexico; luis.pp@pachuca.tecnm.mx; 4 Escuela de Medicina y Ciencias de la Salud, Tecnológico de Monterrey, Avenida Eugenio Garza Sada 2501, Monterrey 64849, NL, Mexico; jadiaze@tec.mx (J.A.D.-E.); eduardofloresvillalba@tec.mx (E.F.-V.); 5 DT3D/CTI, Rodovia Dom Pedro I (SP-65), Km 143,6-Amarais-Campinas, Campinas 13069-901, SP, Brazil; jorge.silva@cti.gov.br

**Keywords:** spring–mass model, stress softening effects (Mullin’s effect), non-Gaussian model, biomaterial residual strains, biological tissues, real-time simulations

## Abstract

In this article, a recent formulation for real-time simulation is developed combining the strain energy density of the Spring Mass Model (SMM) with the equivalent representation of the Strain Energy Density Function (SEDF). The resulting Equivalent Energy Spring Model (EESM) is expected to provide information in real-time about the mechanical response of soft tissue when subjected to uniaxial deformations. The proposed model represents a variation of the SMM and can be used to predict the mechanical behavior of biological tissues not only during loading but also during unloading deformation states. To assess the accuracy achieved by the EESM, experimental data was collected from liver porcine samples via uniaxial loading and unloading tensile tests. Validation of the model through numerical predictions achieved a refresh rate of 31 fps (31.49 ms of computation time for each frame), achieving a coefficient of determination *R*^2^ from 93.23% to 99.94% when compared to experimental data. The proposed hybrid formulation to characterize soft tissue mechanical behavior is fast enough for real-time simulation and captures the soft material nonlinear virgin and stress-softened effects with high accuracy.

## 1. Introduction

Modeling the mechanical behavior of soft biological tissue is a complex task that has derived in the development of a great number of constitutive material models. Some of the material models that have been used in the literature for predicting the nonlinear behavior observed in experimental data are those proposed by Dorftmann and Ogden [1], Holzapfel [2,3,4], Arruda-Boyce [5], Cantournet et al. [6], Elías-Zúñiga et al. [7], and references listed therein. These models consider a different number of material constants that need to be determined using experimental data [8,9]. It is also known that the majority of these constitutive material models are not appropriate to perform real-time simulations and thus, simplified models such as the Spring–Mass Model (SMM) formulation have been developed [10,11,12,13,14]. These SMM formulations are based on the linear elasticity theory and Hooke’s law. Therefore, these are used for small material elongations (*λ* < 10%). The SMM is easy to implement, and computer simulations of the material response are performed in real-time, but its accuracy is lower than that attained by finite element methods. In an attempt to improve the SMM accuracy for larger materials elongations, several approaches have been proposed in the literature. For instance, Chen et al. [10] introduced a novel approach for the SMM considering a nonlinear mass-spring model to predict the global deformation experienced by soft tissue. They found that their proposed nonlinear model provides realistic and faster results compared to those of finite element method (FEM) simulations. A hybrid model that uses boundary element method (BEM) and the SMM was proposed by Zhu and Gu in [11] to simulate the dynamic behavior of soft tissue when interacting with surgical instruments. This model provides accurate visual and haptic feedback in the real-time surgical training system for laparoscopic surgery. Motivated by the low computational cost required by the SMM to simulate in real-time soft tissue changes compared to FEM-based methods, Patete et al. [12] implemented the SMM for predicting breast tissue deformations using an iterative algorithm to estimate the spring’s rest length and stiffness, overcoming critical issues such as the selection of the spring stiffness and the damping factor. It is well-known that SMM is physically correct if one has a relationship between the spring constants and the physical response behavior exhibited by soft materials. Thus, by assuming incompressible and isochoric material behavior, Duan et al. [13] developed a tetrahedral mass-spring formulation that takes into account nonlinear effects for realistic simulation of soft-biological tissue. Then, they implemented a virtual reality environment for laparoscopic cholecystectomy, finding that their model is capable of predicting large tissue deformation in real-time simulations. Regarding the determination of the elasticity parameters of the mass-spring-damper model (lumped element model) of deformable objects, Natsupakong and Çavusoglu [14] used an optimization algorithm that minimizes the matrix norm of the error between the stiffness matrices of the linear lumped element model and of the linear finite element method of the same object. They investigated the accuracy attained by their optimization algorithm considering several test objects in two and three dimensions with triangular, quadrilateral, tetrahedral, and hexahedral elements. They found that quadrilateral and tetrahedral elements predict soft-tissue Young’s modulus value with good accuracy. One must notice that this method is more flexible than existing ones in the literature, since no assumptions on the material elasticity parameters need to be made.

The articles of Delingette et al. [15], Picinbono et al. [16], Holzapfel et al. [17], Ostaja-Starzewski [18], Meier et al. [19], Nealen et al. [20], Luo and Xiao [21], Tang et al. [22], Liu et al. [23], San Vicente et al. [24], Del Castillo et al. [25], Nikolaev [26], Kot et al. [27], Lloyd et al. [28,29], Omar and Zhong [30,31], Nguyen et al. [32], Zhang et al. [33], Dong et al. [34], Va et al. [35], Aryeetey et al. [36], Tripicchio et al. [37], Ballit and Dao [38], and references cited therein, have good discussions on how to incorporate typical biological properties and behavior of soft tissue, and how to perform a correct mapping of the elastic properties among physical objects and the SMM elements. All these related works attempt to simulate soft tissue deformation for surgical real-time visual and force haptic interaction.

It is evident that a lot of work has been done to increase the accuracy and efficiency of the spring–mass models to effectively capture real-time soft tissue deformations. Therefore, in order to improve the efficiency and computational performance of real-time simulations, the study reported in this article proposes a new hybrid formulation based on an equivalent energy spring model (EESM) with a stiffness function that depends on soft tissue deformations. We assume that the strain energy density model formulation is equal to that of a one-dimensional spring that matches with that of a non-Gaussian constitutive material model. Furthermore, we consider in the proposed material model the important softening phenomena associated with the observed real-time mechanical behavior of biological materials at the virgin and softened state assuming that the hyperelastic nonlinear and anisotropic material behavior can be described as an equivalent “isotropized” material [6,7].

## 2. Hybrid Material Model

### Mathematical Formulation

**Figure 1:**
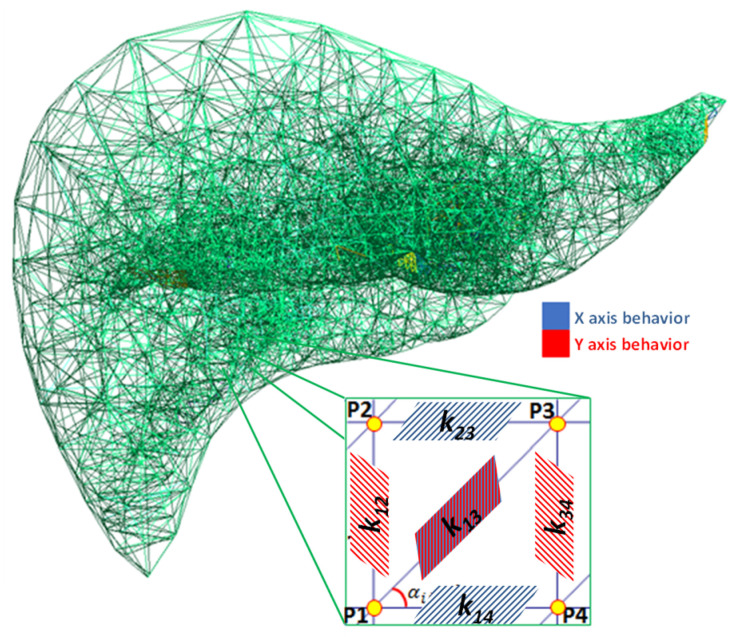
A mesh of springs represented with unidimensional elements using the SMM.

During implementation of the SMM for predicting the response of biological tissue in real-time [12,13], each organ is considered as a construction of mass points and springs, as shown in Figure 1. In this approach, the non-linearity that exhibits soft tissue is obtained by a random combination of the material stiffness assembled with an internal skeleton structure that provided variable stiffness. To calculate the force *F_i_* at a point *P_i_* in the mesh shown in Figure 1, Hooke’s law is used to determine the force of a linear spring that connects *P_i_* with *P_j_* considering the stiffness *k_ij_*, the initial spring length *L_ij_*, and the current spring length at the time of the calculation, using the following expression [13]:

(1) $$F_{i}=k_{ij}\left(\left|P_{j}-P_{i}\right|\right)-L_{ij})\frac{P_{j}-P_{i}}{\left|P_{j}-P_{i}\right|}$$

Equation (1) provides good precision for small tissue deformations. However, the main drawback of this expression is the lack of a physical relationship between the mechanical properties such as the Young modulus and the Poisson ratio with the parameters used to predict the response behavior of soft tissue [12,13]. In order to find the elasticity constants, a subjective trial-and-error calibration process has to be performed to find mesh deformations. In spite of the accuracy attained by the SMM for predicting small tissue deformations, this model cannot predict the material nonlinear response behavior when subjected to large deformations because the mechanical behavior of soft tissue is no longer described by the linear elastic theory. Therefore, there is a need for a new model to capture the material behavior for small or large deformations, while keeping the efficiency of the SMM to perform real-time simulations.

**Figure 2:**
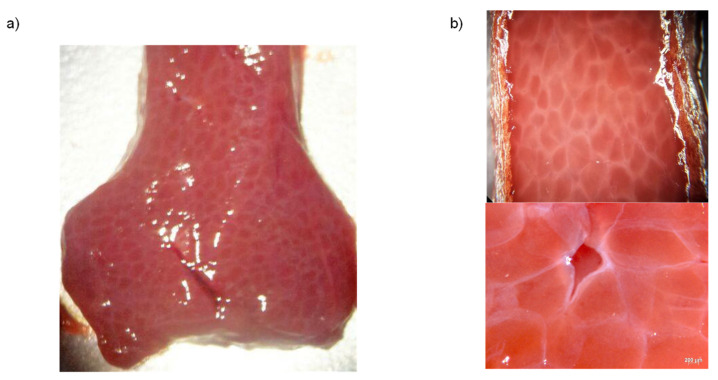
Porcine liver sample showing the histological composition of the tissue: (**a**) Microscopic view of the tissue sample showing its heterogeneous composition; (**b**) Parenchyma cells and fibers structures in a microscopic view of a porcine liver tissue sample, showing the histological composition.

To develop a new material model that captures soft tissue nonlinear effects, one must bear in mind the material histological composition. In fact, one can assume that soft tissue is a composite material formed by a matrix with isotropic behavior and a volume fraction of reticular fibers with a nonlinear anisotropic behavior. Figure 2 illustrates a portion of a porcine liver sample that corroborates our material structure assumptions.

In order to model the behavior of soft tissue and other composite materials, we introduce a new formulation based on the SMM and the non-Gaussian material constitutive model proposed by Elías-Zuñiga in [7].

This constitutive model was selected since it has been applied with success to characterize the mechanical behavior of multiple biological and composite materials. Some of the materials studied include mice skin and tracheal membranes [7], brominated isobutylene and paramethylstyrene copolymer and reinforced natural rubber reinforced with multiwall carbon nanotubes (BIMSM-MWCNT and NR-MWCNT respectively) [39], poly(glycolide-co-caprolactone) and polypropylene sutures [40], and magnetorheological polyurethane elastomers reinforced with carbonyl iron microparticles [41], to name a few. Moreover, the hybrid formulation with SMM proposed in this work allows to perform real-time simulations, opening a whole wide range of potential opportunities.

In the material model introduced by Elías-Zuñiga et al. in [7], they used the rule of mixture to create an equivalent strain energy density (ESED) model, which includes the material energy contributions from isotropic and anisotropic volumetric fractions. Thus, they assumed that the total strain energy density *W_T_* of this ESED model is defined as:

(2) $$W_{T}=\left(1-f\right)W_{iso}+fW_{aniso},$$

where *W_iso_* and *W_aniso_* are, respectively, the isotropic and anisotropic strain energy densities, and *f* represents the equivalent anisotropic volumetric fraction contribution to the total material energy density [42,43,44]. To account for the isotropic contribution (matrix), we use the non-Gaussian strain energy density given as [43]

(3) $$W_{iso}\left(\lambda_{1},\lambda_{2},\lambda_{3}\right)=\mu \left[N\left(\beta \lambda_{r}+ln\left(\frac{\beta }{\sinh\beta }\right)\right)-ln\left(\frac{\beta }{\lambda_{r}}\right)\right]+c,$$

where *λ_r_* is the relative chain stretch defined as

(4) $$\lambda_{r}=\frac{\lambda_{chain}}{\lambda_{L}},$$

$\lambda_{L}=\sqrt{N}$ represents the fully extended chain stretch, *N* is the chain number of chain links of length *l*, *λ_chain_* is the chain stretch given as

(5) $$\lambda_{chain}\equiv \sqrt{\frac{I_{1}}{3}},\;\mathrm{with}\;I_{1}=\lambda_{1}^{2}+\lambda_{2}^{2}+\lambda_{3}^{2},$$

*c* is a constant [43,44,45].

and $\beta =L^{-1}\left(\lambda_{r}\right)$ is the inverse of the Langevin function  L(β) defined as

(6) $$\lambda_{r}=L\left(\beta \right)=\coth\beta -\frac{1}{\beta },$$

which can be written in simplify form using Puso approximation as [46]

(7) $$\beta =\frac{3\lambda_{r}}{1-\lambda_{r}^{3}},$$

For the case of *W_aniso_*, we use the isotropized strain energy density expression discussed in [6,7] that has the form

(8) $$W_{aniso}=\frac{A_{1}}{3}\left(I_{1}-3\right)+\frac{A_{2}}{9}{\left(I_{1}-3\right)}^{2}-\frac{2A_{1}}{3}ln\sqrt{I_{3}},$$

where *A*_1_ and *A*_2_ are the energy density fitting parameters, and $I_{3}=\lambda_{1}^{2}\lambda_{2}^{2}\lambda_{3}^{2}.\;$ Thus, substitution of Equation (3) and Equation (8) into Equation (1) gives the equivalent strain energy density expression that can be used to model soft tissue:

(9) $$W_{T}=\left(1-f\right)W_{iso}+f\left(\frac{A_{1}}{3}\left(I_{1}-3\right)+\frac{A_{2}}{9}{\left(I_{1}-3\right)}^{2}-\frac{2A_{1}}{3}ln\sqrt{I_{3}}\right).$$

Notice that the use of Equation (9) for real-time simulation is restricted by the computational time required to solve this model at each amount of stretch to get the material response behavior. Therefore, a hybrid formulation is proposed based on the Strain Energy Density Function (SEDF) given by Equation (9), and the SMM, which has low computational cost. In this equivalent energy spring model (EESM), we attempt to find an equivalent spring stiffness using an equivalent energy density function; in other words, the equivalence is established with a Neo–Hookean model for one-dimensional spring that is assumed to have variable stiffness value. Here the total strain energy at certain amount of stretch *λ* is considered to be equivalent to the one obtained from the SEDF, at the same amount of stretch, so that the following relationship holds:

(10) $$W_{s}\left(\lambda_{s}\right)=\frac{\sigma \varepsilon }{2}=\frac{F\left(\lambda_{s}-1\right)}{2\nu_{ij}}=\frac{k_{s}\lambda_{s}}{2\nu_{ij}}\left(\lambda_{s}-1\right)=W_{T}\left(\lambda_{1},\lambda_{2},\lambda_{3}\right),$$

where *W_s_* is the total strain energy density of the EESM element, $\nu_{ij}$ is a geometrical parameter that represents the transversal area of the soft tissue object that is used to find the material model parameters, *λ_s_* is the spring element stretch, and *k_s_* is the stiffness function of an EESM element. For uniaxial extension, the total strain energy density *W_T_* of an incompressible material could be found by considering that $\lambda_{1}=\lambda ,\;\lambda_{2}=\lambda_{3}=1/\lambda^{1/2}.$ Therefore, it is possible to find the material parameters for each spring to represent the soft tissue non-linear behavior as a function of the amount of stretch. In this case, the stiffness function is determined from the precalculated equivalent energy considering the SEDF of the isotropic and anisotropic volumetric fractions contributions using Equation (9). Thus, from Equation (10) one can find the relationship that allows to compute the stiffness function of an EESM element:

(11) $$k_{s}\left(\lambda \right)=\frac{2\nu_{ij}\left(\left(1-f\right)W_{iso}+fW_{aniso}\right)}{\left(\lambda_{s}^{2}-\lambda_{s}\right)}.$$

**Figure 3:**
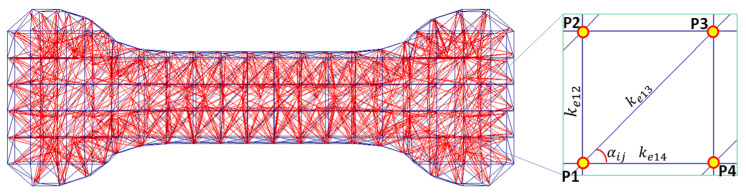
Graphical representation of the creation of mesh of EESM elements that are used for real-time simulation.

This expression helps in finding the element stiffness value that causes a stretched spring to reach the same soft tissue strain energy density at the same amount of stretch. In this case, *k_s_* is calculated for a specific configuration of the EESM element, using the material parameter values obtained from experimental uniaxial extension data. The analysis with the SMM is performed by considering the creation of EESM elements in a simulation mesh. Each EESM element is assumed to be one-dimensional spring that has an initial length equal to the distance between two connected points (*P_i_* and *P_j_*), as shown in Figure 3.

During the implementation of this algorithm, the EESM stiffness function, $k_{ij},$ is used at each stretch value to define a 3D array. This array is pre-calculated based on the parameters that characterized the material mechanical behavior, and by defining a variable stiffness parameter, given by the following expression

(12) $$k_{ij}\left(\lambda_{s}\right)=\left[k_{sx}\left(\lambda_{s}\right),k_{sy}\left(\lambda_{s}\right),k_{sz}\left(\lambda_{s}\right)\right].$$

**Figure 4:**
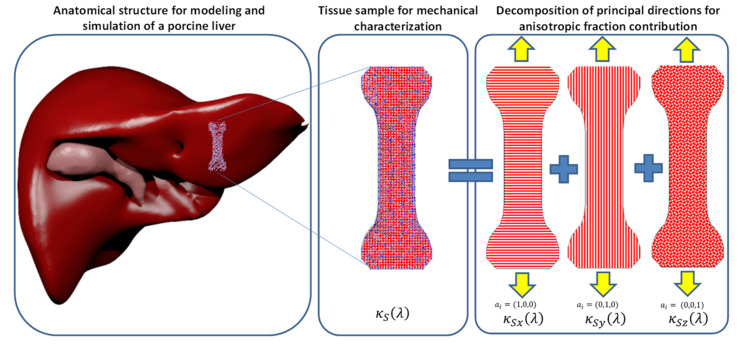
Graphical representation of the uniaxial mechanical test for characterization of soft tissue anisotropic properties.

By using this method, one can find the parameters of a simplified model that allows real-time simulation using the mechanical properties of soft tissue organs collected from uniaxial extension tests. In addition, notice that all the above energy density expressions can be computed by considering the principal stretch fibers direction and those fiber families oriented at 55°, and described by (1,1,1) (and all variations (−1,1,1), (1,−1,1), (1,1,−1), and so on). See [6,7], and references cited therein. Further, we assume that the contribution of the material fibers have random distribution along the principal axes, and that the properties obtained are distributed based on the fiber main orientation. The EESM formulation for uniaxial tension considers that the contribution of the stiffness in the principal directions is the one described in the model and used to predict its mechanical behavior, as shown in Figure 4.

To simplify its calculation, the value of $k_{ij}$ is stored in the initialization phase for each EESM at all values of $\lambda_{s}.$ During the main refresh cycle of the real-time simulation, the method uses the stiffness value, which is calculated at the same elongation experienced by the soft tissue, as the stiffness parameter needed by the EESM.

## 3. Experimental Tests

To characterize the soft tissue mechanical behavior using the EESM, experimental uniaxial tensile tests in porcine liver tissue samples were performed to assess the accuracy attained from our proposed method.

### 3.1. Sample Preparation

**Figure 5:**
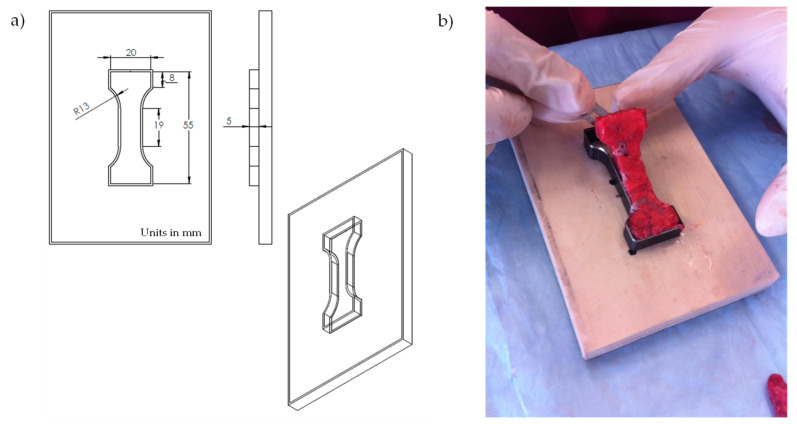
Liver sample preparation to carry out uniaxial loading and unloading cyclic tensile tests. (**a**) Tooling dimensions for cutting liver fresh samples. (**b**) Liver sample geometry.

To perform the experimental tests on the liver tissue, we followed the specimen preparation procedure proposed by Umale et al. [47] and Brunon et al. [48]. However, in our case we decided to cut the liver samples according to the tooling dimensions of 55 × 20 × 5 mm, as shown in Figure 5a,b. All liver samples were cut considering the same general orientation shown in Figure 5b. Then, quasi-static uniaxial experimental tests were carried out on the cut fresh liver samples without preconditioning at room temperature. The tests were performed considering loading and unloading cyclic tests since the softening effects are to be investigated as well. Since soft tissue is an incompressible and inhomogeneous material, it is evident that experimental tests provide information about the material response behavior linked to different fiber volume fraction, fiber orientation, and tissue anisotropic properties.

### 3.2. Experimental Setup

**Figure 6:**
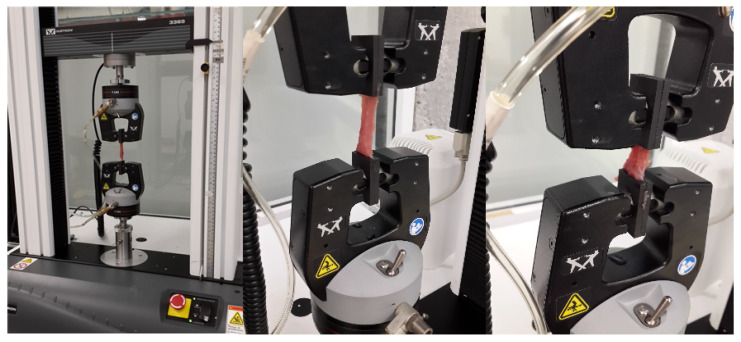
Experimental set up to carry out uniaxial tests in porcine liver tissue samples.

**Figure 7:**
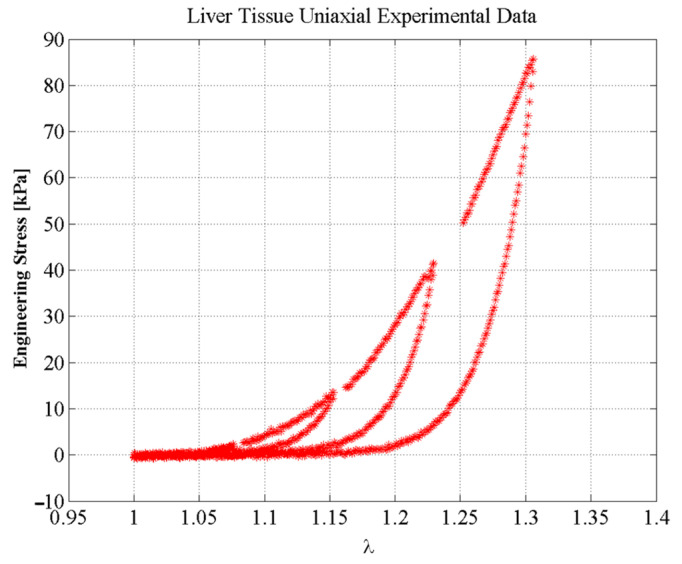
Experimental data for uniaxial tensile test on porcine liver tissue samples.

To collect experimental data needed to compute the stiffness values of the EESM elements given by Equation (11), we performed cyclic loading and unloading uniaxial extension test on porcine liver parenchyma samples. All uniaxial tests were performed in an Instron 3365 electromechanical universal testing machine with load cell of 5 kN, and movable crosshead velocity of 0.01 mm/s, as illustrated in Figure 6. Destructive tests were carried out to identify the liver tissue maximum elongation at break. Then, three loading and unloading cycles were set at different elongation stretch values, as shown in Figure 7.

## 4. Computational Implementation

### 4.1. Simulation Setup

**Figure 8:**
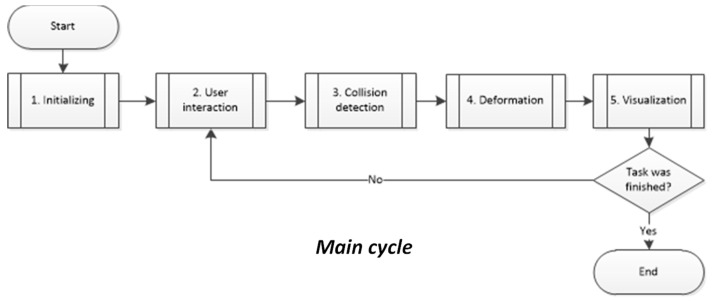
Flow diagram of the main cycle used as a framework for real-time simulation setup.

Figure 8 shows the flow diagram used to implement the EESM material model for getting real-time simulation of soft tissue behavior subjected to deformation fields using Python, Visualization Toolkit (VTK), NumPy, SciPy, Wx, Matlab, and tools such as collision detection, and main interaction cycle for the virtual environment discussed in previous works [49]. This computer algorithms were implemented using average hardware to avoid any bias. The simulations were run in an equipment with an Intel^®^ Core™ i7-4510U @ 2.00 GHz with 8 GB RAM and an NVIDIA GeForce 710 M. All simulations used the same standard mesh, which is a liver geometry composed by 5322 elements.

### 4.2. Model Integration

**Figure 9:**
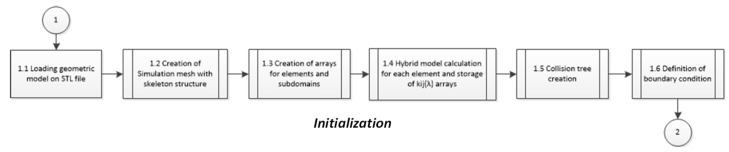
Flow diagram of implementation of proposed model in the initialization phase.

Figure 9 shows the flow algorithm diagram that was followed to calculate the EESM parameters as a function of the organ tissue geometry. In this step, we use the material properties of the organ soft tissue obtained from uniaxial experimental tests.

For the EESM, the strain energy density of a linear spring is obtained from the neo-Hookean model using Equation (10) and equating this value with that obtained from Equation (9) at the same amount of stretch starting from the undeformed state (λ=1) to the maximum specimen stretch value ($\lambda_{max}$), and by considering incremental stretch step values Δλ. The computed value of the SEDF is stored in the program initialization phase. Then, for an elongation λ, one can compute the value of $k_{s}\left(\lambda \right)$, which allows to obtain the soft tissue energy density value at the same amount of stretch.

**Figure 10:**
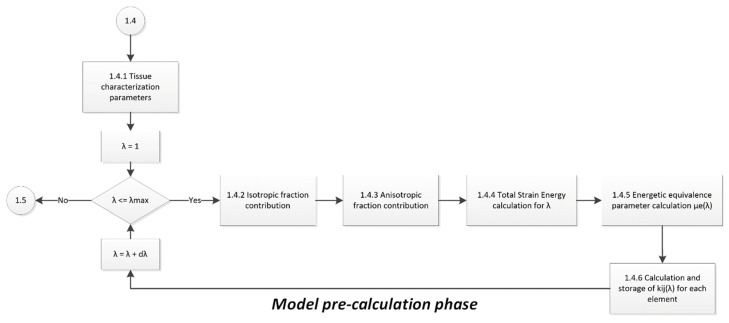
Flow diagram of the implementation of the EESM pre-calculation phase.

Figure 10 shows the flow diagram computer algorithm used to find the EESM value for each spring–mass element considering the soft tissue material constants obtained via uniaxial tests, which aids in calculating the $k_{ij}$ value, which is in storage with the corresponding stretch value experienced by each element.

## 5. Material Constitutive Equation

To find the stress versus stretch material models that will aid in investigating the accuracy of our proposed hybrid model for soft tissue, we take the derivative of Equation (9) with respect to the amount of stretch [44] to get the following Cauchy stress-stretch virgin material constitutive equation

(13) $$T=\left(1-f\right)ℵB+\frac{2f}{3}\left(A_{1}+\frac{2A_{2}}{3}\left(I_{1}-3\right)\right)B-\frac{2fA_{1}}{3}1-p1,$$

where *T* is the Cauchy stress, ***B*** is the left Green-Cauchy deformation tensor defined as

(14) $$B=\lambda_{1}^{2}e_{11}+\lambda_{2}^{2}e_{22}+\lambda_{3}^{2}e_{33},$$

$\lambda_{i}$ are the principal stretches in a common orthonormal frame $\varphi =\left\{O;e_{k}\right\},\;e_{jk}=e_{j}⊗e_{k},\;e_{i}$ are the orthonormal principal directions, *p* is a hydrostatic pressure, and *ℵ* is the soft tissue response function defined as

(15) $$ℵ=\frac{\mu }{3\lambda_{r}}\left[\beta +\frac{1}{N_{8}}\left(\frac{1}{\lambda_{r}}-\frac{1}{\beta \left(1-\lambda_{r}^{2}-\frac{2\lambda_{r}}{\beta }\right)}\right)\right].$$

Stress softening and residual strains can be predicted using the following material model [50,51,52]:

(16) $$\tau_{k}=\left(\left(1-f\right)ℵ\lambda_{k}^{2}+\frac{2f}{3}\left(A_{1}+\frac{2A_{2}}{3}\left(I_{1}-3\right)\right)\lambda_{k}^{2}-\frac{2fA_{1}}{3}-p\;+\frac{\mu \lambda_{k}}{2C}g_{k}\left(\lambda_{1},\lambda_{2},\lambda_{3}\right)\right)e^{-b\sqrt{\left(M-m\right)\left(\frac{m}{M}\right)}},$$

*k* = 1, 2, 3 (no sum) with

(17) $$g_{k}\left(\lambda_{1},\lambda_{2},\lambda_{3}\right)=\frac{\partial \overset{3}{\underset{a=1}{\sum }}{\left(\lambda_{\max\;a}^{n}-\lambda_{a}^{2}\right)}^{2}}{\partial \lambda },$$

where *C* is a material constant, *n* is a fitting parameter that in general takes the value of 1, *b* is a dimensionless softening parameter, $\lambda_{a}$ are the principal stretches, and $\lambda_{\max a},$ a=1,2,3 are the maximum stretch values at which unloading begins on the primary loading path.

From Equations (16) and (17), one can show that the Cauchy stress-stretch constitutive virgin material model is given as:

(18) $$T_{j}-T_{k}=\left(\left(1-f\right)ℵ+\frac{2f}{3}\left(A_{1}+\frac{2A_{2}}{3}\left(I_{1}-3\right)\right)\right)\left(\lambda_{k}^{2}+\lambda_{k}^{2}\right),$$

and for the stress-softened material as

(19) $$\tau_{j}-\tau_{k}=\{\left(\left(1-f\right)ℵ+\frac{2f}{3}\left(A_{1}+\frac{2A_{2}}{3}\left(I_{1}-3\right)\right)\right)\left(\lambda_{j}^{2}-\lambda_{k}^{2}\right)+\;\frac{\mu \lambda_{k}}{2C}\left(\lambda_{j}g_{j}\left(\lambda_{1},\lambda_{2},\lambda_{3}\right)-\lambda_{k}g_{k}\left(\lambda_{1},\lambda_{2},\lambda_{3}\right)\right)\}e^{-b\sqrt{\left(M-m\right)\left(\frac{m}{M}\right)}},$$

where *j ≠ k* = 1, 2, 3 (no sum). The value of $\lambda_{r}$, *m*, and *M* can be computed by considering that for simple extension homogeneous deformation state, $\lambda_{1}=\lambda ,\;\lambda_{2}=\lambda_{3}=\lambda^{-1/2}.$ This gives the following relationships

(20) $$\lambda_{r}=\sqrt{\frac{1}{3N}(\lambda^{2}+2\lambda^{-1})},\;m=\sqrt{\lambda^{4}+2\lambda^{-2}},\;M=\sqrt{\lambda_{\max}^{4}+2\lambda_{\max}^{2}}.$$

Finally, using

(21) $$\sigma =TF^{-1},\;\mathrm{and}\;\sigma_{s}=\tau F^{-1},$$

we can compute the engineering stress-stretch constitutive material equations for the virgin and for the stress-softened materials, respectively.

## 6. Results

**Figure 11:**
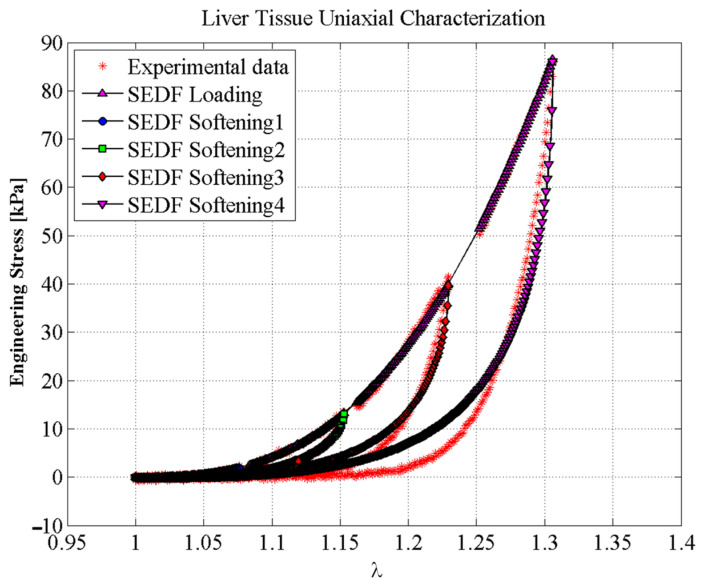
Predictions of the mechanical behavior of porcine liver tissue using the SEDF. The material constants used to fit experimental data were: *μ* = 0.1 kPa, *N* = 1.1974, *b* = 3.3389, *A*_1_ = 9.6754 kPa, *A*_2_ = 7543.6 kPa, *C* = 9.5273 kPa, and *f* = 0.2755.

To address the accuracy attained from our proposed hybrid material model, we use uniaxial loading and unloading experimental data collected from porcine liver samples [49]. Figure 11 shows the predicted loading and unloading engineering stress curves obtained from Equations (18) and (19). One can see from Figure 11, that the material model based on the equivalent SEDF captures experimental data well. In this case, the material constants used to fit experimental data were: *µ* = 0.1 kPa, *N* = 1.1974, *b* = 3.3389, *A*_1_ = 9.6754 kPa, *A*_2_ = 7543.6 kPa. *C* = 9.5273 kPa, and *f* = 0.2755. To find the stiffness function of an EESM element, the SEDF is computed using Equation (9) with $\nu_{ij}=3.569\times 10^{-5}$ m^2^.

**Figure 12:**
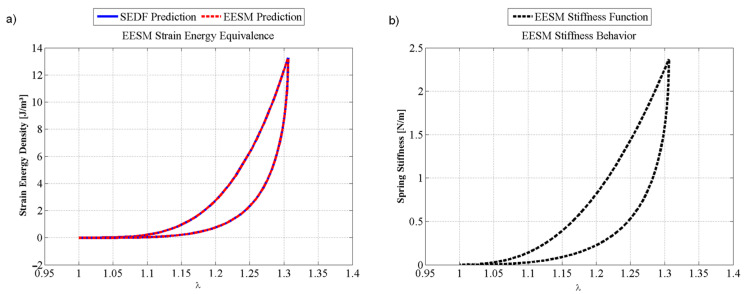
EESM results: (**a**) strain energy equivalence between SEDF and EESM; (**b**) stiffness function of an EESM element that predicts the behavior of the porcine liver tissue, based on the experimental data and the SEDF equivalence. The material constants used to fit experimental data were: *μ* = 0.1 kPa, *N* = 1.1974, *b* = 3.3389, *A*_1_ = 9.6754 kPa, *A*_2_ = 7543.6 kPa, *C* = 9.5273 kPa, and *f* = 0.2755.

Figure 12a shows the estimated strain energy density curves attained for the liver samples maximum stretch. Then, the spring stiffness function is determined from Equation (11) so that the SEDF and the EESM have the same value, as illustrated in Figure 12a, while Figure 12b shows the variation of the sample stiffness as a function of the amount of stretch. These stiffness vs. stretch values are storage in a computer subroutine so that this information can be used to investigate in real-time, the material response behavior when subjected to a certain amount of deformation.

**Figure 13:**
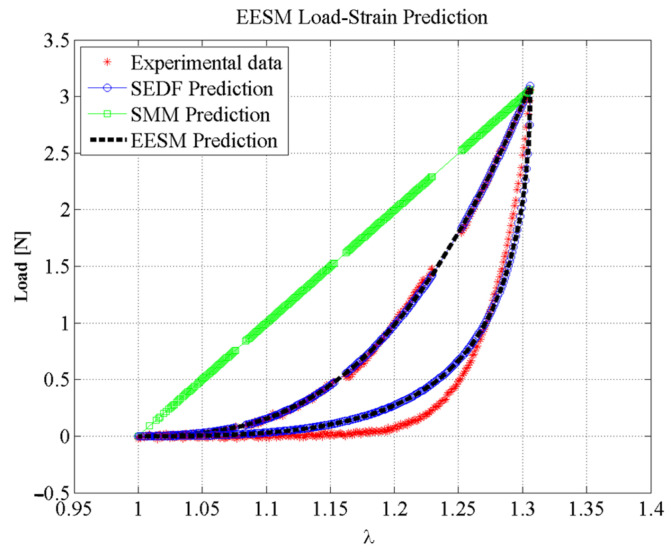
Comparison between the experimental data of porcine liver tissue in uniaxial tensile test, the SEDF prediction, a simple SMM prediction, and the proposed EESM prediction.

Figure 13 shows the experimental data and the SEDF predictions compared to the SMM and EESM element predictions using the computed stiffness values. Both models were evaluated under the same conditions, using the same software and hardware, and estimating the best fitting stiffness parameter values to represent the material stress-stretch behavior. Notice that the EESM proposed approach predicts the soft tissue nonlinear behavior and the Mullins effects well.

**Figure 14:**
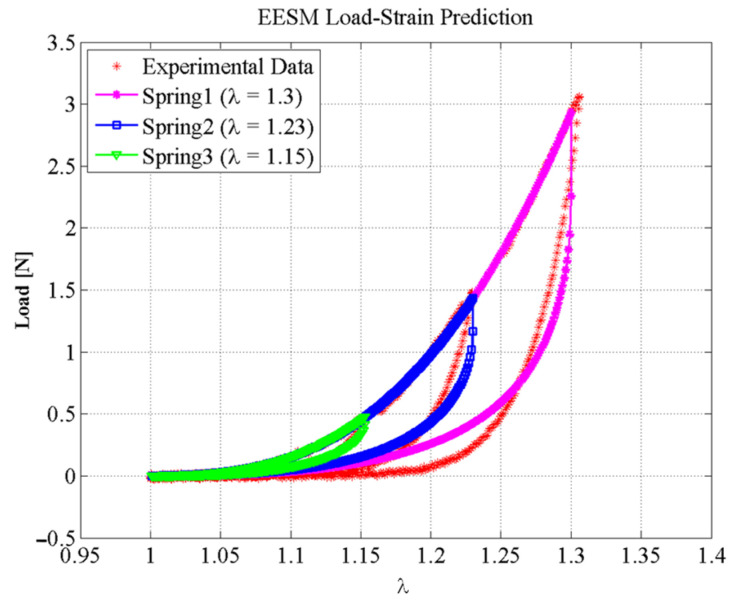
Predictions of the mechanical behavior of three different EESM elements representing springs with variable stiffness that characterize the behavior of porcine liver tissue.

In addition, three springs with different initial and final lengths (called Spring1, Spring2, and Spring3) were used to run some numerical tests. Spring1, Spring2, and Spring3 were elements subjected to the maximum stretch value of λ=1.3, λ=1.23, and λ=1.15, respectively. Predictions obtained from these springs are shown in Figure 14. Notice that theoretical predictions and experimental data agree well, which is an indication of the accuracy achieved from our proposed approach.

### Simulation Performance

For validation purposes, a simulation test environment was created as discussed in Section 4, to allow interaction with the meshes and for simulating soft tissue behavior. The accuracy attained from our proposed model was assessed using the coefficient of determination (R-squared, *R*^2^) that helps to identify if the majority of the experimental data have been correctly predicted by the EESM and SMM models [53]. For the experimental data shown in Figure 10, the estimated values of *R*^2^ using the prediction values computed from EESM were 99.94% and 93.23% for the loading and unloading curves, respectively. However, the *R*^2^ value for the predicted SMM values when compared to experimental data was about 15.11%. This is an indication of the limitations of the SMM for predicting soft-tissue nonlinear behavior, as shown in Figure 13.

In addition predictions from the EESM formulation are obtained in an initialization phase that does not need to be computed in real-time since it does not influence the graphical rendering refresh cycle. Then, the stiffness functions are stored for all EESM elements in the simulation mesh. Notice that the performance in real-time no longer depends on the constitutive model based on the SEDF however, it is a function of the simulated geometry mesh, and of the number of assumed EESM elements.

**Figure 15:**
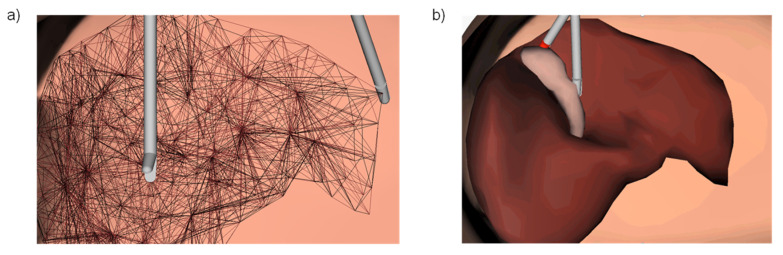
Real-time simulation of mechanical behavior of a liver geometry using EESM elements: (**a**) Internal mesh of one-dimensional elements representing springs with variable stiffness in function of the constitutive model; (**b**) shaded visualization of the deformation mesh calculated on real-time with 31 fps.

In the validation tests, the implementation of the proposed model gets a refresh rate of 25 to 32 Hz. An initial test was done with the liver geometry, shown in Figure 15, conformed by 5322 EESM elements requiring 1522 ms for initialization process. Each simulation cycle for calculation of deformation and graphical update needed 31.49 ms, representing a 31-fps update rate. The computer used for this test has the following CPU: Intel^®^ Core^TM^ i7-4510U @ 2.00 GHz/8 GB RAM.

**Figure 16:**
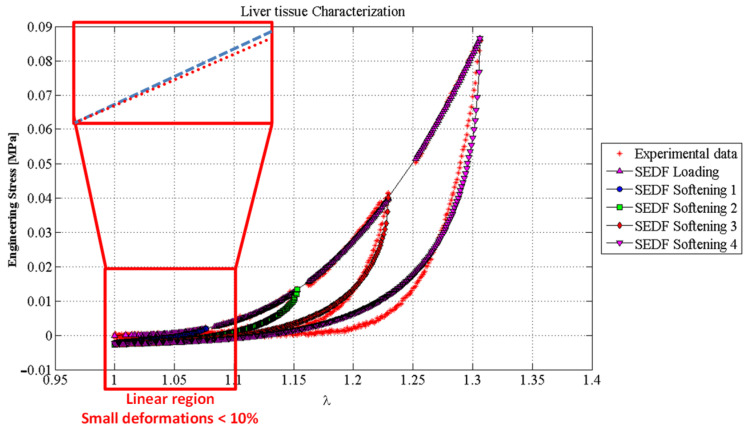
Graphical description of why linear elastic models like SMM have issues predicting the mechanical properties of soft tissues and biological materials.

**Figure 17:**
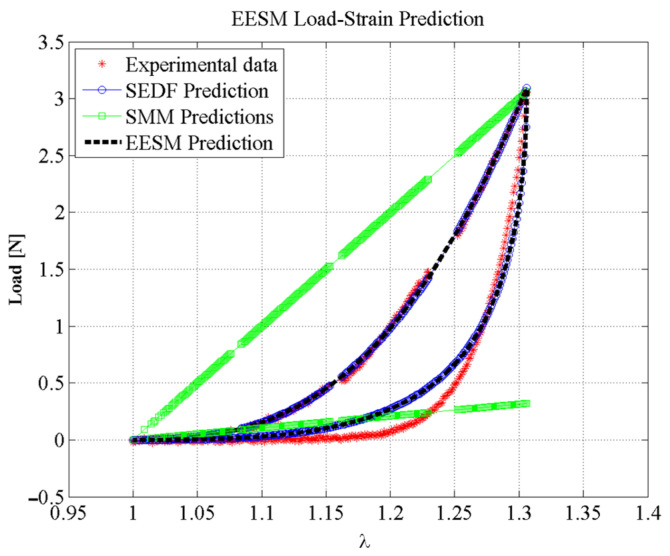
Comparison of the load-strain predictions obtained using the SMM versus the EESM, demonstrating that the EESM formulation can represent the behavior of soft tissue and biological materials in large deformation, including characterization of the nonlinear behavior and the Mullin’s effect.

In summary, material models based on linear material behavior such as the SMM approach can predict with good accuracy the behavior of soft tissue only for small deformations (lower than 10%) because of the material linear response, as illustrated in Figure 16. The accuracy of the EESM approach to predict soft-tissue behavior at larger deformations (bigger than 10%) when compared to the SMM approach and other linear elastic models is clearly seen in Figure 17 therefore, it is concluded that our proposed hybrid model for predicting nonlinear material behavior observed in soft biological tissue works well.

## 7. Discussion

The results shown in the previous section demonstrate that it is possible to use the proposed EESM approach to predict in real-time, the mechanical behavior of soft tissue. This model has the benefits of the two main components of the hybrid formulation: (1) fast computational time response of the SMM; (2) the accuracy to predict the material mechanical behavior of the SEDF that uses the isotropized strain energy density function. The EESM formulation demonstrated its capacity to represent mechanical properties of soft tissue in real-time simulations. The construction between the SEDF and the SMM shows good results as a hybrid model, being able to have the accuracy of a complex constitutive model with the computational performance of a simple model. The low error attained by our proposed model in predicting soft tissue behavior for loading and unloading real-time simulations indicates that our solution procedure works well for describing nonlinear phenomena typical of biological tissues such as stress-softened effect. In fact, Mullin’s effect is not taken into account in the soft-tissue SMM published in the literature. To the best of the author’s knowledge, this is the first publication that includes Mullin’s effect in biological tissues real-time simulations.

It is evident that our proposed approach can be improved if soft tissue effects such as viscoelastic properties and the rigidity and topology of multiscale mechanical networks are considered. It is well-known that network topology of soft-tissue can be described with the loss modulus (the imaginary part of the complex shear modulus G*) by the fractal material network dimension [54]. This idea is confirmed in a recent work published by Leggett et al. [55], in which they found that epithelial cells organize into fractal-like clusters that exhibit a branched architecture with fractal dimension predicted by diffusion-limited aggregation. In other words, the moving cells organize into multicellular clusters that promote tissue formation and wound healing, to mention a few. Therefore, one must consider the important role that mathematical modelling has on describing the performance of soft tissue when subjected to certain types of stimuli. Recently, scientist have used fractional and fractal calculus to describe phenomena that ordinary derivatives cannot predict, such as the anatomical network structure or the dynamical physiologic networks that are fractals. See, for instance, the works published by West et al. [56], Beleanu et al. [57], Beleanu et al. [58], and Ameen et al. [59]. Therefore, and in order to enhance our proposed material hybrid model to reproduce living anatomy or physiology of the human body for real-time medical simulations, we need to use fractal representation of the expressions that provide the Cauchy stress-stretch expressions. In this sense, we recall that the isotropic part of the Cauchy stress-stretch virgin material expression (13) is derived from an expression of the form

(22) $$T_{i}=\lambda_{i}\frac{\partial W}{\partial \lambda_{i}}-p,\;i=1,2,3\;\mathrm{no}\;\mathrm{sum}.$$

Since living cells and organisms are forced to adopt self-organizing fractal structures [59], the fundamental laws of physics needs to be modeled using fractal derivatives to be able to capture the qualitative and quantitative real-time system behavior. In this sense, Equation (22) must be cast into an expression of the form

(23) $$T_{i}=\lambda_{i}\frac{\partial^{\alpha }W}{\partial \lambda_{i}^{\alpha }}-p,\;i=1,2,3\;\mathrm{no}\;\mathrm{sum},$$

where *α* is a fractal dimension whose value depends mainly on tissue anatomy, physiology, and on the distribution of the strain energy-density. The mathematical treatment of Equation (23) can be done considering the solutions procedures discussed in [57,58,59,60]. Recently, other fractal theory definitions [61,62], and solution methods have been proposed [63,64,65,66]. However, the discussion of our findings using fractal material constitutive equations shall be addressed in a forthcoming paper to be published somewhere else.

Finally, to model soft tissues other than liver using Equation (11), one needs to perform experimental uniaxial tests to get the equation parameter values to predict in real-time the corresponding tissue haptic behavior.

## 8. Conclusions

The material constitutive model proposed in this study (equivalent energy spring model, EESM, with a stiffness function that depends on the material’s amount of stretch) combines the computational efficiency of the spring–mass model with the accuracy of non-Gaussian material model to predict, in real-time, the nonlinear behavior of soft tissue.

In this model, the material strain energy density is assumed equal to that of a one-dimensional spring that matches with that of a non-Gaussian constitutive material model. Furthermore, the anisotropic behavior exhibited by soft tissue materials was captured by an equivalent “isotropized” material model that allows to find the parameter values of each neo-Hookean spring that emulates soft tissue non-linear behavior as a function of the amount of stretch.

The computed value of the SEDF was stored in the program initialization phase to ease the computation of the material stiffness to obtain, for the same amount of stretch, the corresponding soft tissue energy density value. Then, this information was used to investigate in real-time, the material response behavior when subjected to loading and unloading uniaxial deformation state. Theoretical predictions obtained via the proposed EESM confirmed the capacity of the material model in predicting soft tissue nonlinear behavior with good accuracy since the *R*^2^ values were 99.94% for loading tests, and 93.23% for unloading data. This represents an improvement of about 75% when compared to the theoretical values obtained from the spring–mass model. In summary, the proposed hybrid model predicts loading and stress-softened soft tissue behavior well and it has the computational performance of a spring–mass model.

This article sheds new light on getting in real-time the response of soft tissue materials, considering not only the material nonlinear characteristics exhibited at large deformations, but also computational aspects such as precision and performance to simulate a virtual reality environment for surgical laparoscopic training by having accurate visual and haptic feedback.

## Data Availability

Not applicable.
